# Molecular association of glucose-6-phosphate isomerase and pyruvate kinase M2 with glyceraldehyde-3-phosphate dehydrogenase in cancer cells

**DOI:** 10.1186/s12885-016-2172-x

**Published:** 2016-02-24

**Authors:** Mahua R. Das, Arup K. Bag, Shekhar Saha, Alok Ghosh, Sumit K. Dey, Provas Das, Chitra Mandal, Subhankar Ray, Saikat Chakrabarti, Manju Ray, Siddhartha S. Jana

**Affiliations:** Department of Biological Chemistry, Indian Association for the Cultivation of Science, Kolkata, 700032 India; Cancer Biology and Inflammatory Disorder Division, Indian Institute of Chemical Biology, Kolkata, 700032 India; Department of Biochemistry and Biophysics, Texas A&M University, College Station, TX 77843-3474 USA; 13 Regent Estate, Kolkata, 700 092 India; Structural Biology and Bioinformatics Division, Indian Institute of Chemical Biology, Jadavpur, Kolkata, 700 032 India; Department of Biophysics, Bose Institute, Kolkata, India

**Keywords:** Glyceraldehyde-3-phosphate dehydrogenase, Glucose-6-phosphate isomerase, Pyruvate kinase M2, Molecular association, Malignancy

## Abstract

**Background:**

For a long time cancer cells are known for increased uptake of glucose and its metabolization through glycolysis. Glyceraldehyde-3-phosphate dehydrogenase (GAPDH) is a key regulatory enzyme of this pathway and can produce ATP through oxidative level of phosphorylation. Previously, we reported that GAPDH purified from a variety of malignant tissues, but not from normal tissues, was strongly inactivated by a normal metabolite, methylglyoxal (MG). Molecular mechanism behind MG mediated GAPDH inhibition in cancer cells is not well understood.

**Methods:**

GAPDH was purified from Ehrlich ascites carcinoma (EAC) cells based on its enzymatic activity. GAPDH associated proteins in EAC cells and 3-methylcholanthrene (3MC) induced mouse tumor tissue were detected by mass spectrometry analysis and immunoprecipitation (IP) experiment, respectively. Interacting domains of GAPDH and its associated proteins were assessed by *in silico* molecular docking analysis. Mechanism of MG mediated GAPDH inactivation in cancer cells was evaluated by measuring enzyme activity, Circular dichroism (CD) spectroscopy, IP and mass spectrometry analyses.

**Result:**

Here, we report that GAPDH is associated with glucose-6-phosphate isomerase (GPI) and pyruvate kinase M2 (PKM2) in Ehrlich ascites carcinoma (EAC) cells and also in 3-methylcholanthrene (3MC) induced mouse tumor tissue. Molecular docking analyses suggest C-terminal domain preference for the interaction between GAPDH and GPI. However, both C and N termini of PKM2 might be interacting with the C terminal domain of GAPDH. Expression of both PKM2 and GPI is increased in 3MC induced tumor compared with the normal tissue. In presence of 1 mM MG, association of GAPDH with PKM2 or GPI is not perturbed, but the enzymatic activity of GAPDH is reduced to 26.8 ± 5 % in 3MC induced tumor and 57.8 ± 2.3 % in EAC cells. Treatment of MG to purified GAPDH complex leads to glycation at R399 residue of PKM2 only, and changes the secondary structure of the protein complex.

**Conclusion:**

PKM2 may regulate the enzymatic activity of GAPDH. Increased enzymatic activity of GAPDH in tumor cells may be attributed to its association with PKM2 and GPI. Association of GAPDH with PKM2 and GPI could be a signature for cancer cells. Glycation at R399 of PKM2 and changes in the secondary structure of GAPDH complex could be one of the mechanisms by which GAPDH activity is inhibited in tumor cells by MG.

**Electronic supplementary material:**

The online version of this article (doi:10.1186/s12885-016-2172-x) contains supplementary material, which is available to authorized users.

## Background

An important characteristic of rapidly proliferating malignant cells is their capacity of high aerobic glycolysis. Cancer cells display a high level of glucose uptake and enhanced lactic acid production as compared to normal cells, a phenomenon widely known as the Warburg effect [1]. Glyceraldehyde-3-phosphate dehydrogenase (GAPDH, EC 1.2.1.12) is a highly conserved and important enzyme for catalyzing the 6th step of glycolysis by conversion of glyceraldehyde-3-phosphate (GAP) and inorganic phosphate (Pi) into 1, 3-bisphosphoglycerate (1, 3-BPG) in the presence of NAD^+^. GAPDH has been shown to exhibit a wide variety of cellular functions like apoptosis, neurotransmission, phagocytosis, and vesicle fusion in ER to Golgi transport etc., besides its role as a glycolytic enzyme [2–6]. Interestingly, GAPDH can also translocate to the nucleus. Induction of GAPDH translocation to nucleus causes cell death in human SH-SY5Y neuroblastoma and rat neonatal cardiomyocytes [7, 8] and lowering such nuclear localization suppresses apoptosis in ovarian cancer cells [9]. Recent studies have established enhanced expression of both mRNA and protein level of GAPDH in human lung cancer tissues, pancreatic adenocarcinomas, and dunning rat prostate cancer cell lines compared with normal tissues and cell lines [10–12]. High expression of GAPDH has been correlated with upregulation of cell cycle related genes that are involved in G2/M transition, M phase regulation, glycolytic genes, and down regulation of gluconeogenesis in non-small cell lung carcinoma [13].

GAPDH is a homotetrameric protein with molecular mass of 145 kDa. Each identical subunit contains 333 residues with molecular mass of 35.9 kDa [14]. In contrast, Bagui et al. [15] and Patra et al. [16] showed that GAPDH is a heterodimer consisting of two non identical subunits approximately of 33 and 55 kDa when purified from Ehrlich ascites carcinoma (EAC) cells, mouse sarcoma tissue, and human leukemic leukocytes. Interestingly, methylglyoxal (MG), a normal metabolite could inactivate GAPDH activity in a wide variety of malignant cells and tissues, whereas it has no effect on GAPDH from normal sources [17]. However, the mechanism behind MG mediated inhibition of GAPDH activity in cancer cells is unknown.

Here, we report for the first time that purified GAPDH complex purified from EAC cells contains two distinct 55 kDa subunits associated with a 33 kDa subunit. We identify these two 55 kDa subunits as two glycolytic enzymes- one is GPI and another is PKM2. GAPDH can interact with both of these enzymes in EAC cells as well as in 3MC induced tumor tissue. We establish that MG glycates R399 residue located in M2 insert of PKM2 and changes the secondary structure of the GAPDH complex. This study indicates a different molecular association of GAPDH and underscores the glycation events by methylglyoxal in cancer cells.

## Methods

### Propagation of EAC cells

EAC cells were maintained and grown in the peritoneal cavity of Swiss albino mice as described previously [18] following the guidelines of the Institutional Animal Ethics Committee of Indian Association for the Cultivation of Science (IACS) and ARRIVE guideline for reporting animal research. Guidelines were approved by the Institutional Animal Ethics Committee of IACS. Approximately, 10^6^ cells were diluted in 0.2 ml of sterile normal saline (0.9 %) and were used for inoculums. The cells were harvested after 10–15 days. Erythrocytes were removed by washing with 0.45 % NaCl.

### Mass spectrometry

GAPDH was purified from EAC cells based on its enzymatic activity which was checked after each step of purification as described previously [15]. Purified GAPDH complex from EAC cells was run on 7.5–15 % SDS-PAGE, and bands were visualized by Coomassie blue stainining. Bands were excised from the gel and processed for mass spectrometry analysis separately. Briefly, after reduction, alkylation, and digestion with In-Gel tryptic Digestion Kit (Thermo Fisher Scientific, Waltham, MA, USA), the resulting peptide mixture was spotted using α-cyano-4-hydroxy cinnamic acid (5.0 mg/mL, Sigma) in 70 % acetonitrile in 0.1 % TFA and analyzed by MALDI-TOF/TOF (Applied Biosystem 4700, USA) mass spectrometer in reflector mode for protein identification by peptide mass fingerprint [19]. The mass spectrum was acquired using 4000 series explorer v3.5 software and peak list was generated between 800 and 4000 Da with a signal-to-noise ratio of 25. The searching parameters used for identification of acquired peptide spectrum were taxonomy: mouse; cleavage enzyme: trypsin; maximum number of miss cleavage 1; mass tolerance 100 ppm; peptide charge: +1; variable modification: oxidation of methionine and carbamidomethylation of cysteine, partial N-terminal acetylation, modification of glutamine as fixed modification of peptides. Data was analysed using GPS explore v3.0 (TM) software. The combined MS and MS/MS results were matched with the NCBInr, SWISS PORT and MSDB databases using MASCOT software v2.1 in order to identify the proteins. Proteins were identified on the basis of significant MASCOT Mowse-score (*p* < 0.05 where p is the probability that the observed match in a random event).

The quality and accuracy of the data were examined by determination of false discovery rate (FDR) values using Mascot software (Matrix Science, London, UK) as previously reported [20]. Briefly, Mascot generic format (MGF) file of raw combined MALDI-TOF and MALDI-TOF/TOF data were created using following parameters (MS peak filter: Peak density filter: 65/200 Da; S/N: 10; No of peaks: 200; Area: 50; MS/MS peak filter: Peak density filter: 65/200 Da; S/N: 5; No of peaks: 200; Area: 20). MGF files were submitted to Mascot website to calculate the FDR values of respective proteins. To find out the stable glycation product, we treated purified protein complex with 1 mM MG for five days at room temperature [21]. Samples were run on 7.5–15 % SDS-PAGE followed by mass spectroscopy analysis.

### Development of tumor in the hind leg of mice

All animal experiments were carried out after receiving the approval of guidelines from the Institutional Animal Ethics Committee. Guidelines were adhered to ARRIVE for reporting animal research. Guidelines were approved by the Institutional Animal Ethics Committee. Mouse tumor was generated using a previously reported method [22]. Chemical carcinogen, 3MC, (Sigma-Aldrich, St. Louis, Missouri, USA) was injected intramuscularly into the left hind leg of Swiss albino female mice for three times with one week intervals. Each dose was 10 mg.kg^−1^ bodyweight in 0.05 ml of olive oil. At 98–105 days, full grown tumor was developed at the site of 3MC injection in left leg whereas no tumor formation was visible in contra lateral right leg.

### Electrophoresis, immunoblot analysis and immunoprecipitation

Mouse tissue and EAC cell extracts were prepared in modified radio immunoprecipitation assay buffer (RIPA, composed of 50 mM Tris ⁄ HCl of pH 8.0, 150 mM sodium chloride, 1.0 % Nonidet P-40, 4 mM EDTA of pH 8.0) with 1 mM dithiothreitol, 0.5 mM phenylmethylsulfonyl fluoride and 1 % protease inhibitor cocktails (Sigma-Aldrich) at 4 °C as previously reported [22, 23]. Briefly, mouse tissue and EAC cell were homogenized in RIPA buffer and centrifuged at 10, 000 g for 10 min. The supernatant was fractionated by 8 % SDS-PAGE and then transferred to polyvinylidene difluoride membranes. The membranes were blocked in PBS containing 5 % BSA and 0.05 % Tween-20 for 1 h, and incubated overnight at 4^0^ C with primary antibodies to GAPDH (1:4 000), GPI (1:4 000, Santa Cruz Biotechnology Inc., CA, USA), PKM2(1:3 000; Cell Signaling Technology, Danvers, MA, USA) or β-tubulin (1:4 000; Sigma-Aldrich). The membranes were then washed and incubated with horseradish peroxidase-conjugated secondary antibodies against mouse or rabbit IgG at room temperature for 2 h and developed with the supersignal west femto reagent (Thermo Fisher Scientific). Chemiluminescence signal was captured on Kodak film. Relative band intensity was quantified by using ImageJ software (NIH, Bethesda, MD, USA) after normalizing band intensity with β-tubulin.

For immunoprecipitation, tissue supernatants were incubated with specific primary antibodies for overnight at 4 °C. Primary antibodies were then pooled down by incubating with protein G agarose for an additional 4 h at 4 °C. Immunocomplexes were then subjected to western blot analysis as described previously. To detect GPI and PKM2 in the immunoprecipitate by western blot analysis, we used Veriblot IP (Abcam, Cambridge, UK) secondary antibody which detects only primary antibody used for western blot, but not for immunoprecipitation.

### Enzymatic assay

Both EAC and mouse tissue extracts in RIPA buffer were used for enzymatic assay. GAPDH activity was assayed in Triethanolamine-HCl buffer, pH 8.5 as described previously [15]. Briefly, 1 ml of assay mixture contained 50 mM triethanolamine buffer, 50 mM Na_2_HPO_4_ (Sigma-Aldrich), 0.2 mM EDTA, 0.5 mM NAD^+^ and 0.04 mM of D-glyceraldehyde- 3-phosphate (Sigma-Aldrich). The reaction was carried out by the addition of 5 μg extracts, and monitored by recording absorbance of NADH at 340 nm at 30 s intervals. Increase in the absorbance values remained almost linear for 3 min (ΔA: 0.025–0.040 min^−1^). One unit of activity of GAPDH was defined as the amount of enzyme required to convert 1 μmol of NAD^+^ to NADH per min under standard assay conditions. The specific activity was calculated as units of activity present per mg of protein. For MG mediated inhibition study, 30 μg of protein from 3MC induced tumor tissue and EAC cell lysates were preincubated with 0–1 mM MG for 10 min at 25 °C, and enzymatic activity of GAPDH and GPI was measured. We followed resorcinol method developed by Roe et al. [24] to measure GPI activity. Briefly, resorcinol detects the keto group present in fructose-6-phosphate. Samples from 3MC induced tumors and EAC were incubated in 50 mM Tris ⁄ HCl buffer of pH 7.4 with 1 mM of substrate glucose-6-phosphate in presence and absence of MG at different concentration for 10 min. 1 g/100 ml resorcinol solution and 10 N HCl at 1:7 ratio were added to the reaction and kept for 10 min at 80 °C. Absorbance at 520 nm of was recorded using Varioskan Flash Elisa Reader (Thermo Fisher Scientific). Absorbance of MG alone was subtracted from that of resorcinol-fructose condensation product. Percent of GAPDH or GPI activity was calculated by considering specific activity of untreated sample as 100.

### Molecular in silico docking analysis

Three-dimensional (3D) structures of human GAPDH (Protein Data Bank (PDB) code: 1U8F, chain O) [25], PKM2 (PDB code: 1ZJH, chain A) and GPI (PDB code: 1JLH, chain A) [26] were collected from the PDB database [27]. 3D co-ordinates of GAPDH - PKM2 and GAPDH - GPI were further utilized for blind docking approach using three different protein-protein docking programs, ClusPro [28, 29], PatchDock [30] and SwarmDock [31], respectively. Top 30 docking solutions from each of the program’s output were selected and were further utilized to identify the interacting interface using the PDBePISA server [32]. Residues involved in the docking interface from each protein chain were identified using in-house perl scripts. Domain involvement of a protein for a docking complex is assigned if 60 % or more residues from the N or C terminal domains are involved in forming the interface. Frequency of hydrogen bonding, salt bridge formation and change in free energy (∆G) of interaction were extracted from the interface features calculated by the PDBePISA [32] program.

## Results

### PKM2 and GPI are associated with GAPDH in cancer cells

**Fig. 1:**
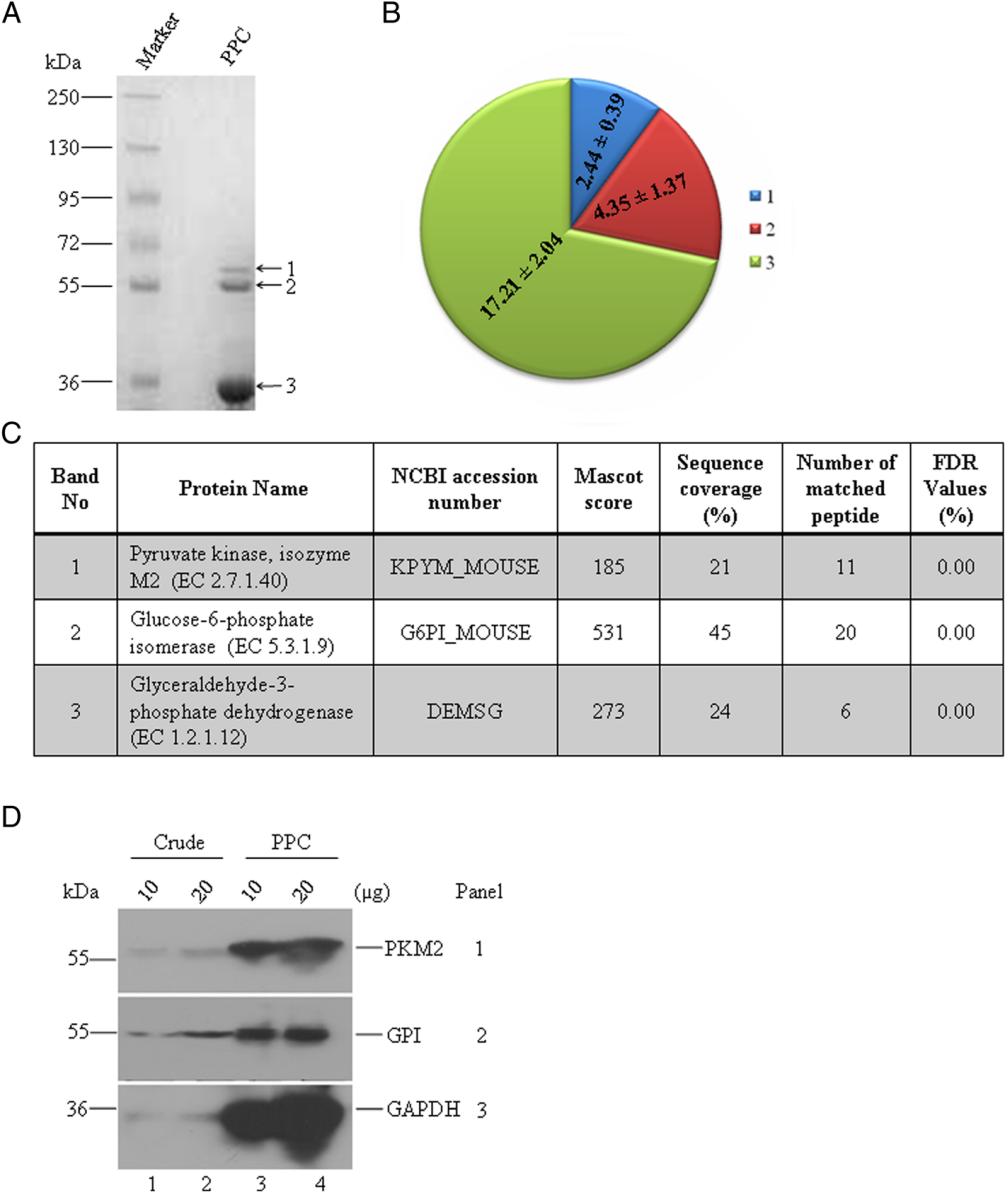
GAPDH is associated with PKM2 and GPI. **a** A representative of coomassie blue stained 7.5–15 % Tris-glycine SDS-PAGE gel of 10 μg purified protein complex (PPC) from EAC cells. Three major bands with different molecular weight approximately 58, 55, 33 kDa are labeled with 1, 2, and 3, respectively. **b** Mole of a subunit in the complex was calculated using a formula = [{(intensity of the band/total intensity of three bands) x loading amount}/molecular mass]. Molar ratio is shown in pie chart. **c** Three bands were cut out from the gel for trypsin digestion followed by MALDI-TOF/TOF analyses. Score of identified proteins from each band is tabulated. Note that bands 1, 2 and 3 contain mainly PKM2, GPI and GAPDH respectively. Data from one representative experiment is shown here. Experiment was repeated six times. **d** Immunoblots of two different amounts of crude extract and purified protein complex (PPC) from EAC cells with antibodies specific for PKM2 (panel 1), GPI (panel 2) and GAPDH (panel 3). Note that both PKM2 and GPI were co-purified with GAPDH

Previous report by Bagui et al. [15] suggested that, on purification and molecular mass determination of GAPDH of EAC cells, 55 kDa subunit is associated with 33 kDa subunit of GAPDH. To characterize this unknown subunit, we purified GAPDH from EAC cells, based on its enzymatic activity (Additional file 1: Figure S1A-B). Additional file 1: Figure S1B shows that specific activity of GAPDH reached approximately 475 units/mg of protein, after passing through DEAE-Sephacell column. Interestingly, when the Sephacell column eluents were subjected to gradient SDS-PAGE, three bands of different sizes of approximately 58, 55 and 33 kDa were visible with different intensities (Fig. 1a). We quantified the band intensity using ImageJ analysis and found that three polypeptides were co-purified at a 2.44 : 4.35 : 17.21 molar ratio, respectively (Fig. 1b), suggesting that two types of polypeptides are associated with GAPDH at different amount in the purified protein complex of EAC cells.

To characterize the subunits associated with GAPDH during purification, we carried out MALDI-TOF/TOF experiments followed by peptide mass fingerprint (PMF) analysis of the three bands present in SDS-PAGE. Figure 1c shows the significantly identified proteins- PKM2 (from band 1), GPI (from band 2), and GAPDH (from band 3). We also performed false discovery rate (FDR) analysis to examine the quality and accuracy of the identified proteins. FDR values indicate that three proteins were identified with zero false positive identification. Sequences of trypsin digested fragments of the three individual bands were mapped with the NCBI database sequences of mouse PKM2 (KPYM_MOUSE), GPI (G6PI_MOUSE), and GAPDH (DEMSG). We found that sequence coverage for PKM2, GPI, and GAPDH were 21 %, 45 %, and 24 % respectively (Additional file 1: Figure S1C-E). A list of proteins detected from individual band in denatured condition is enlisted in Additional file 1: Table S1-3. Detection of PKM2 and GPI by MALDI-TOF/TOF analysis prompted us to confirm these three proteins by immunoblot analysis. Figure 1d shows the detection of three glycolytic enzymes in the DEAE-Sephacell column eluents using specific antibody against each of these enzymes. Note that the amount of each enzyme increases in the eluents (*lanes 3 and 4*) compared with the crude lysates (*lanes 1 and 2*). Taken together, these results suggest that GAPDH isolated from EAC cells is associated with two glycolytic enzymes, PKM2 and GPI.

### Expression of PKM2 and GPI increases in tumor tissue

**Fig. 2:**
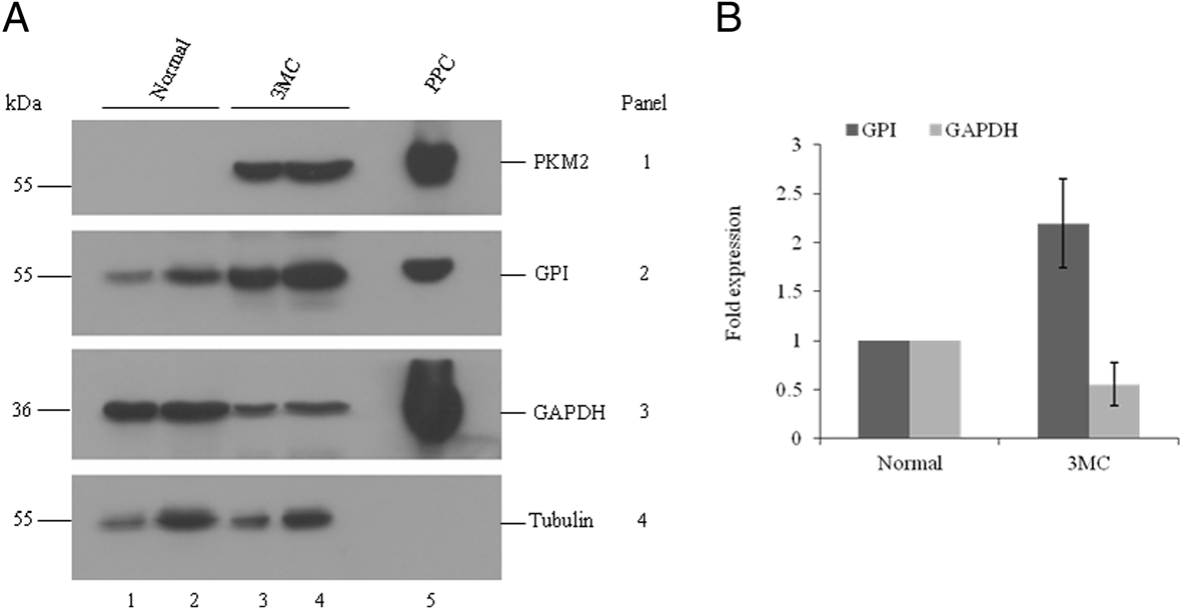
Expression profile of three enzymes in mouse normal and 3MC induced tumor tissues. **a** Lysates were subjected to immunoblot analysis using anti-PKM2 (panel 1), −GPI (panel 2), −GAPDH (panel 3) and β-tubulin (panel 4) antibodies. β-tubulin was used as loading control for comparison of GAPDH, PKM2 or GPI expression between normal (lane 1 and 2) and tumor (lane 3 and 4) tissues. Note that the expression level of PKM2 and GPI is increased in tumor tissue. Purified protein complex (PPC) from EAC cells was considered as positive control for GAPDH, GPI, and PKM2 antibodies (lane 5). **b** Quantification of band intensity of the immunoblot containing GPI and GAPDH. Fold induction in each case was calculated considering the value relative band intensity for normal as “1”. Results are expressed as means ± SD from three independent experiments. **p < 0.01 for GPI or PKM2 in tumor vs normal

To address the question whether this interaction of GAPDH with PKM2 and GPI occurs in other type of cancer cells, we used an in vivo tumor model system, in which tumor was developed in mice muscle by injecting a carcinogen, 3MC. We, first, checked the expression profile of these three glycolytic enzymes in 3MC induced tumor tissue by immunoblot analysis, and compared with that of the normal tissue from the contra lateral leg of same mouse. We found that both PKM2 and GPI were increased whereas GAPDH was reduced in the tumor (*lane 3 and 4*) compared with the normal tissue (*lane 1 and 2,* Fig. 2a). We quantified the expression of each of the enzyme and Fig. 2b shows that expression of GPI is higher by 2.2 ± 0.45 fold whereas as that GAPDH is lower by 1.8 ± 0.22 in 3MC induced tumor compared with normal tissue. On the other hand, PKM2 was not detectable in normal tissue. Christofk et al. [25] have recently shown that PKM2, but not PKM1 (another alternative spliced isoform of PKM), is advantageous for tumor cell growth and critical for tumorigenesis. We checked the expression of PKM1 in 3MC induced tumor tissue. Additional file 1: Figure S2 shows that PKM1 is detectable only in normal tissue but not in the 3MC induced tumor tissue, suggesting that tumor tissue expresses only PKM2.

**Fig. 3:**
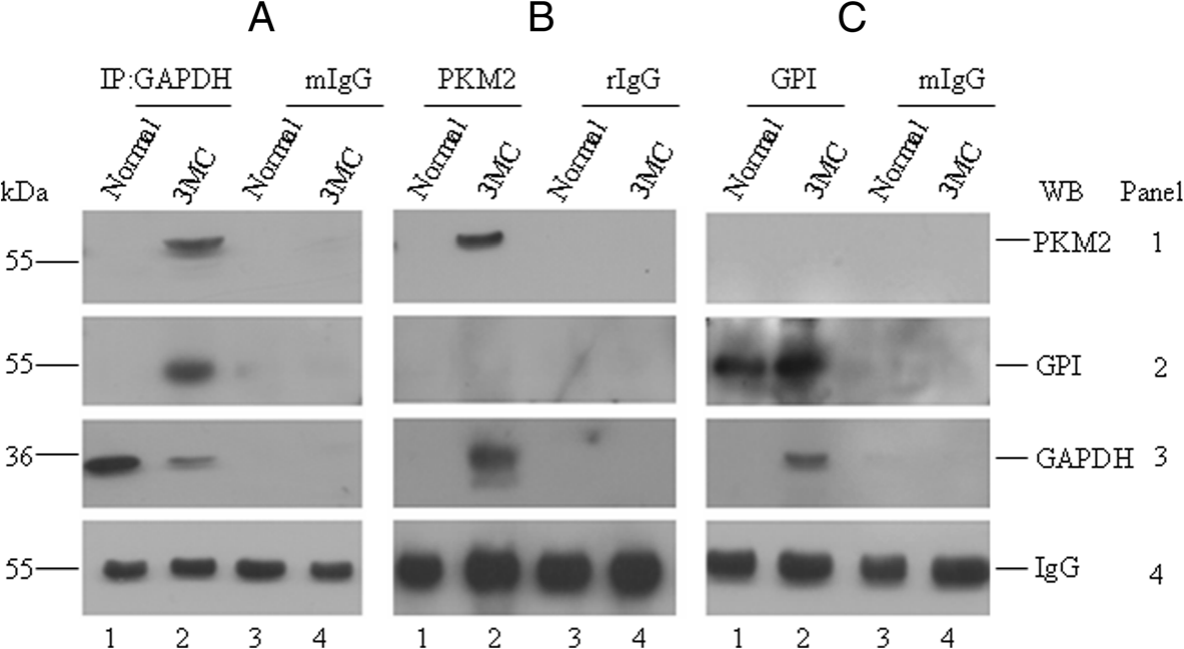
Both PKM2 and GPI interact with GAPDH. **a** Immunoprecipitate of GAPDH antibody was subjected to immunoblot with PKM2 (panel 1), GPI (panel 2), GAPDH (panel 3) or mouse IgG heavy chain (panel 4) specific antibody. **b** Immunoprecipitate of PKM2 was subjected to immunoblot with PKM2, GPI, GAPDH or rabbit IgG heavy chain specific antibody. **c** Immunoprecipitate of GPI was subjected to immunoblot with PKM2, GPI, GAPDH or mouse IgG heavy chain specific antibody. Note that PKM2 was not immunoprecipitated with GPI antibody and vice versa. Mouse IgG was used as negative control for immunoprecipitation of GAPDH and GPI antibodies, and rabbit IgG for PKM2 (lanes 3 and 4). Antibodies against heavy chain of mouse IgG and rabbit IgG were used as loading controls for immunoblots (panel 4)

Association of GAPDH with PKM2 and GPI in tumor cell was validated by immunoprecipitation assay. We immunoprecipitated GAPDH in normal and 3MC induced tumor tissue lysates using antibody against GAPDH, and the precipitate was further probed with antibodies against PKM2, GPI, and GAPDH. In Fig. 3a (and Additional file 1: Figure S3A-B), panels 1 and 2 show that both PKM2 and GPI are detectable in the immunoprecipitate of GAPDH antibody, but not of mouse IgG, in 3MC induced tumor tissue (*lane 2*). Panel 3 (Fig. 3a) indicates that GAPDH is immunoprecipitated specifically by GAPDH antibody, but not by mouse IgG, suggesting that both PKM2 and GPI are associated with GAPDH in tumor but not in normal tissue.

Also, we immunoprecipitated PKM2 in 3MC induced tumor tissue lysate, and probed with antibodies against GAPDH and GPI. Figure 3b shows that only GAPDH (panel 3), but not GPI (panel 2), is detectable in the immunoprecipitate of PKM2, suggesting that PKM2 interacts with GAPDH, but not with GPI. Furthermore, PKM2 was undetectable in the immunoprecipitate of GPI antibody in the 3MC induced tumor tissue lysate (Fig. 3c, panel 1, *lane 2*), confirming that there was no interaction between GPI and PKM2 in tumor cells. Mouse or rabbit IgG was used as control antibody for immunoprecipitation. Immunoblot with secondary antibody against mouse or rabbit IgG but not primary antibody was used as a loading control for immunoprecipitate samples. Altogether these data suggest that tumor cells show increased expression of PKM2 and GPI, and two types of GAPDH association-one is GAPDH-GPI and another is GAPDH-PKM2-exist in the 3MC induced tumor tissue.

### C-terminal domain of GAPDH interacts with PKM2 and GPI

**Fig. 4:**
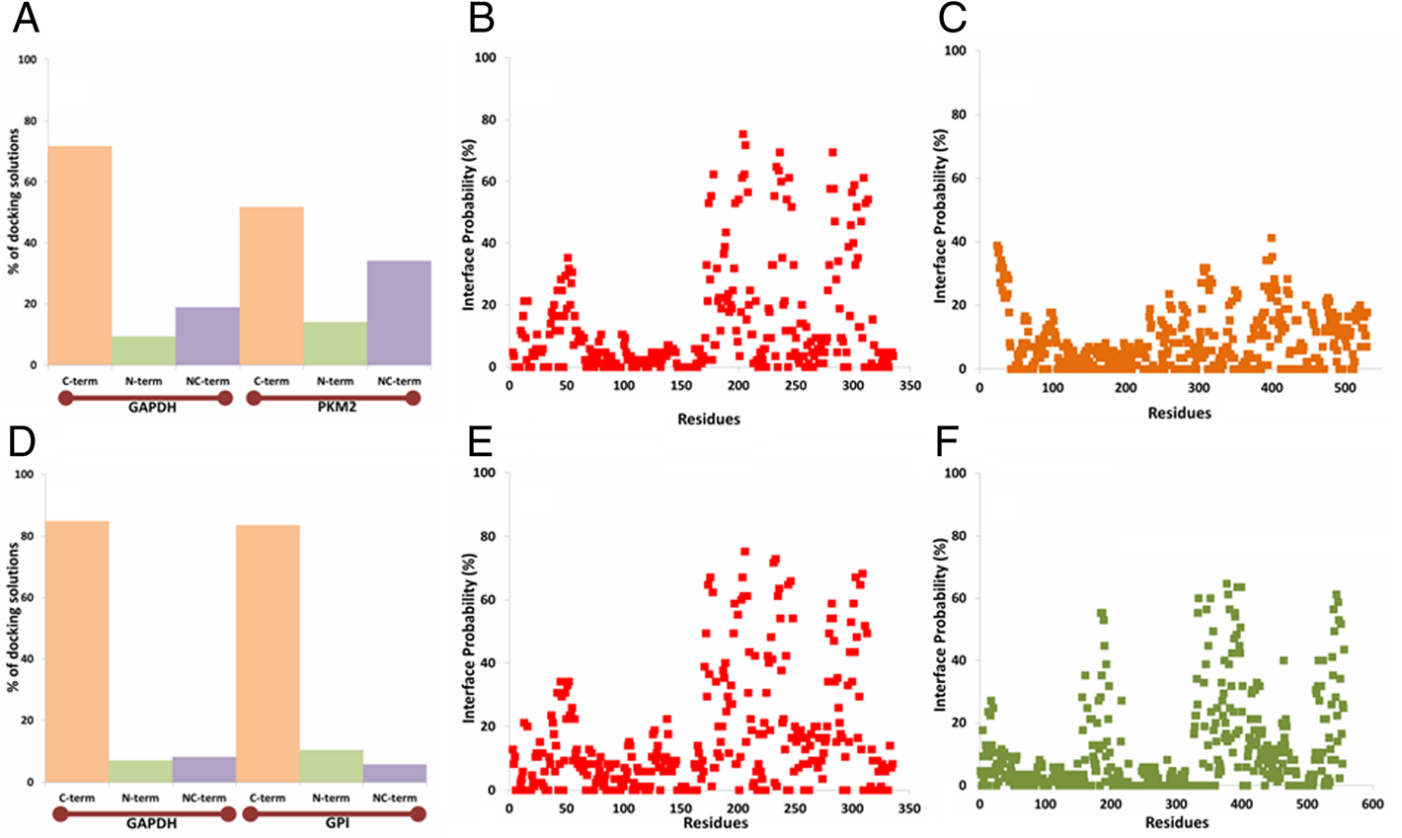
Panel **a** provides the overall occurrence frequencies of C terminal, N terminal, and both NC of GAPDH and PKM2 proteins at the interface observed within the 90 top scoring docking complexes obtained from three different programs whereas panels **b** (GAPDH) and **c** (PKM2) provides the occurrence frequency of each residue within the GAPDH-PKM2 docking complexes. Panels **d**-**f** represent similar domain and residue occurrence frequencies observed within the GAPDH-GPI docking complexes. An interface is regarded as C or N terminal interface if 60 % of the interface residues for each protein reside within C or N termini, respectively

In the preceding section, we have shown that GAPDH can interact with GPI and PKM2 in cancer cells. In order to assess which domain (s) of GAPDH, PKM2 and GPI are involved in interaction; we carried out *in silico* molecular docking analysis. 3D structure of human GAPDH (PDB code: 1U8F, chain O) was docked onto PKM2 (PDB code: 1ZJH, chain A) and GPI (PDB code: 1JLH, chain A) independently without providing any prior information to the docking programs. Top docking solutions from each programs ClusPro [28, 29], PatchDock [30] and SwarmDock [31] were screened and pooled together for interface analysis. Figure 4 and Additional file 1: Figure S4 plot the overall and average frequencies of N or C terminal domain/residue involvement of GAPDH, PKM2 and GPI proteins within the GAPDH-PKM2 (Fig. 4 and Additional file 1: Figure S4A-C) and GAPDH-GPI (Fig. 4 and Additional file 1: Figure S4D-F) docking complexes, respectively. Frequencies of C terminal domain of GAPDH are significantly higher in GAPDH-PKM2 (Fig. 4b) and GAPDH-GPI (Fig. 4e) docking complexes, advocating the role of C terminal part of GAPDH in interaction with both PKM2 and GPI. Similarly, C terminal domain of GPI (Fig. 4f) is more likely to be used in interaction with GAPDH. However, in case of PKM2, it is not quite evident which domain is more preferred to interact with GAPDH despite of the slightly higher abundance of C terminal domain at the interface (Fig. 4c).

### Methylglyoxal can inhibit GAPDH activity in both EAC and 3MC induced tumor

**Fig. 5:**
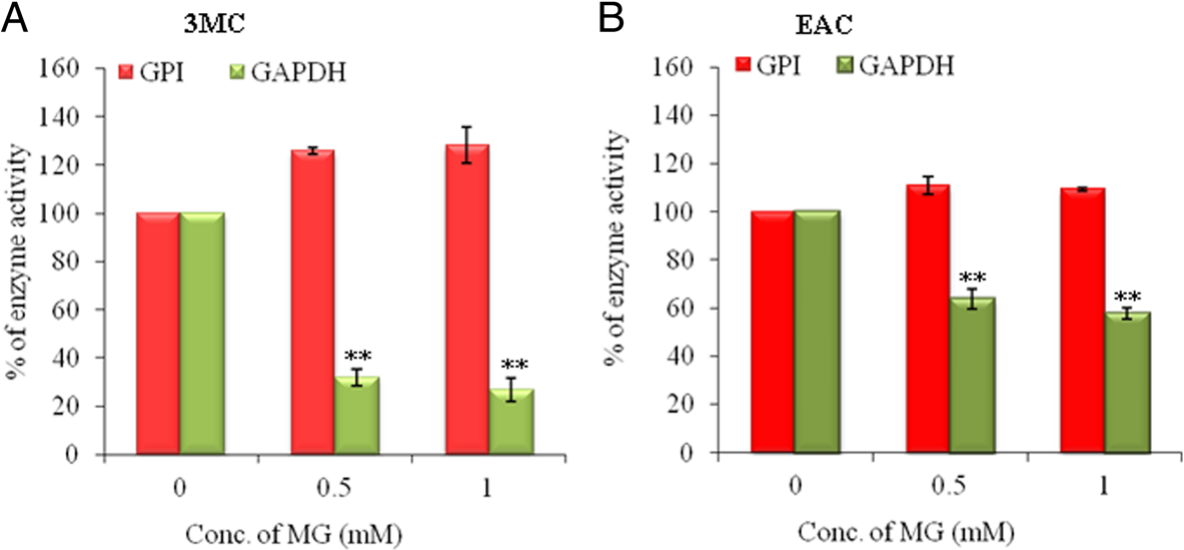
MG inhibits GAPDH activity. Enzymatic activity of GPI and GAPDH in the extracts of 3MC induced tumor tissues (**a**) and EAC cells (**b**). Activity of both enzymes was measured in the absence and presence of 0.5 and 1 mM MG at 25 °C for 10 mins. Percent inhibition was calculated considering the activity of enzyme in absence of MG as 100 % (vehicle). Data presented as means ± SD from three independent experiments. **p < 0.01 vehicle vs 0.5 mM or 1 mM MG

Ray et al. [17] showed that MG inhibits the activity of GAPDH of malignant cells but not of normal cells and benign tumor cells. We found that GAPDH is associated with two other glycolytic enzymes: PKM2 and GPI. Whether PKM2 or GPI activity is affected by MG in tumor cells, we measured the enzymatic activity of all three enzymes in EAC and 3MC induced tumor tissue in the presence of MG. Figure 5 shows that GPI activity is less likely to be inhibited by 1 mM MG in 3MC induced tumor (Fig. 5a) and EAC (Fig. 5b). In contrast, GAPDH activity was reduced to 26.8 ± 5 % in the 3MC induced tumor tissue (Fig. 5a) and to 57.8 ± 2 % in EAC cell lysates (Fig. 5b) of total activity in the presence of 1 mM MG. We could not measure the effect of MG on the activity of pyruvate kinase (PK) in tumor cells, which may be explained due to very low activity of PK in tumor cells [33]. Additional file 1: Figure S5A shows that PK has 0.41 ± 0.12 U/mg and 1.55 ± 0.46 U/mg specific activity in 3MC and EAC, respectively, compared with 12.2 ± 2.4 U/mg in normal tissue. We checked whether MG has any influence on the interaction of these subunits present in 3MC induced tumor in mouse. Additional file 1: Figure S5B shows that both PKM2 and GPI are detectable in the immunoprecipitate of GAPDH antibody in the presence of 1 mM MG (lane 2). Whether the inactivation by MG is due to conformational change of GAPDH complex, we carried out circular dichroism (CD) spectroscopy in the UV range with purified protein complex from EAC cells. CD spectra of untreated GAPDH complexes show two well minima at 208 and 222 nm, which are characteristic of proteins containing α-helical secondary structures. When these GAPDH complexes were treated with MG the minima values at these two wave lengths decreased in a dose dependent manner (Additional file 1: Figure S5C). We quantified the change of helicity in the presence of MG, and Additional file 1: Figure S5D shows that purified GAPDH complex exhibits 19 % and 20 % change in the helicity in the presence of 1 mM MG for 6 h at 208 and 222 nm, respectively. Taken together, these data suggest that MG may inhibit GAPDH activity by changing the helicity of GAPDH complexes (with PKM2 or GPI) without interfering the interaction with them.

### Methylglyoxal glycates M2 insert of PKM2 enzyme

**Fig. 6:**
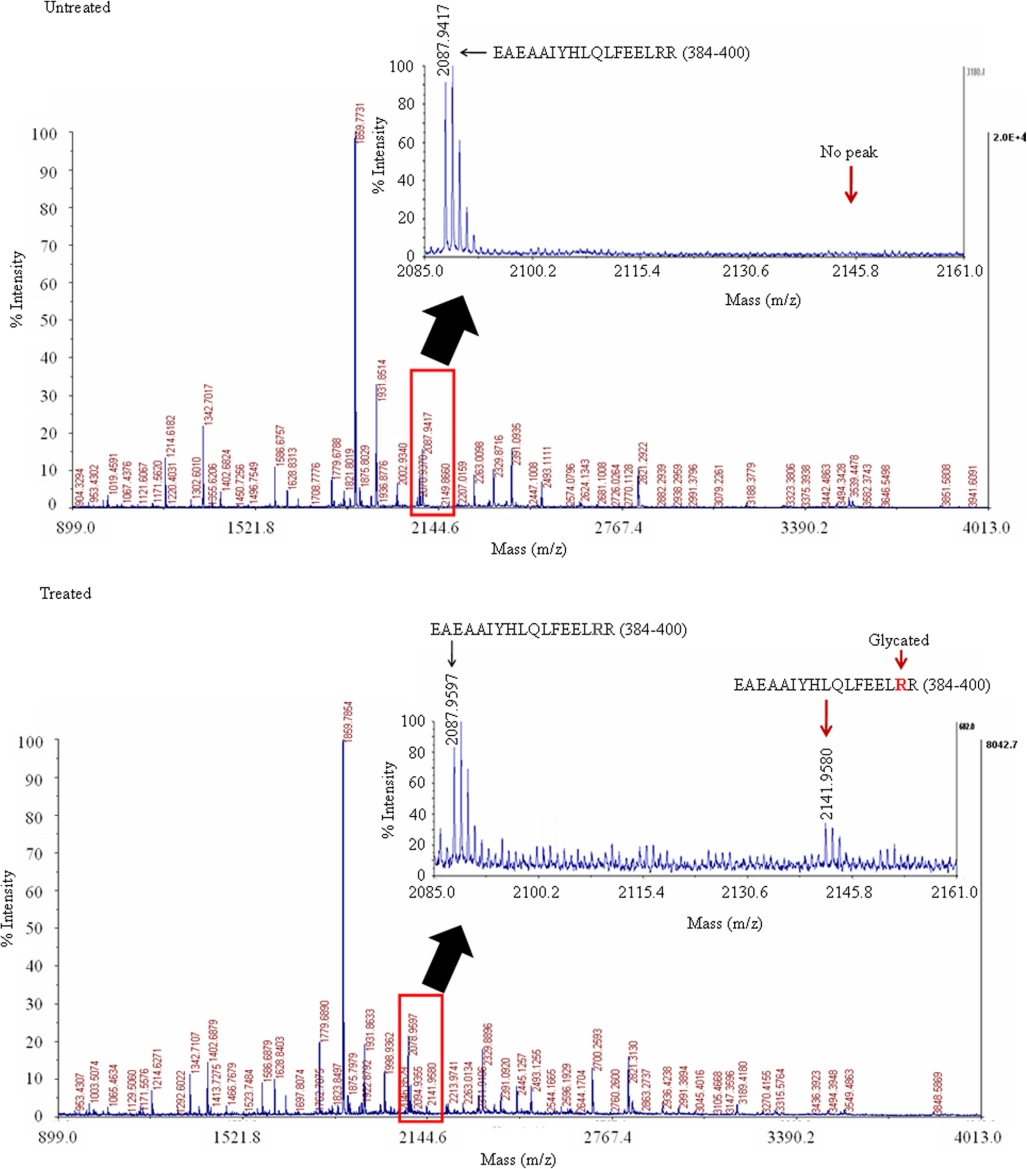
MG glycates M2 insert of PKM2 enzymes. Purified protein complex from EAC was treated with 1 mM MG for 5 days at room temperature. MG treated or untreated samples were run on 7.5–15 % SDS-PAGE gel followed by mass spectroscopy analysis for PKM2. Area nearby glycated peptide’s peak is magnified. Red arrow shows glycation at Arg 399 residue in M2 insert of PKM2 whereas black arrow for nonglycation of the peptide of 17 aa

It is known that MG can glycate arginine and lysine residues in proteins and produce methylglyoxal advanced glycation end-products (MAGE) [19]. Using mass spectrometry analysis we checked if incubation by MG results in any glycation on arginine and lysine residues of GAPDH complex. After incubation with 1 mM MG, bands correspond to PKM2, GPI and GAPDH were analyzed. Due to the mass increase characteristic of a MAGE, glycated peptides had no match in the databases and were therefore rejected [19]. This information can be used to identify the molecular location of specific MAGE in target protein(s). In the mass spectrometry of MG treated proteins, several new peaks appear that do not have predicted m⁄ z values. These new peaks may be due to the miscleavage of peptide(s). To know the mass of probable peptides due to miscleavage, we performed a theoretical digestion with the known protein sequence, considering upto four trypsin miscleavages, cystine alkylation with iodoacetamide and mono-isotopic peptide mass using Expasy website [19]. With these theoretical peptide masses, the mass increment due to a specific MAGE modification (54, 80 and 144 Da for arginine, 72 Da for lysine) were added to find out if there were any new peaks which were matched with the peak of mass spectrometry of MG treated proteins. Using this approach, no such modified peptide peaks were found in case of mass spectrometry of MG treated GAPDH (except an unknown peak at *m/z* 2700.2021, labeled with an arrow) or GPI compared with untreated one (Additional file 1: Figure S6 A-B). Surprisingly, when the mass spectrometry peaks of PKM2 were analyzed (Fig. 6), we found that one arginine residue was modified by MG in the form of hydroimidazolone (MG-H1) (red arrow, observed mass 2141.9580 Da, corresponding to PKM2 peptide 384–400 with *m/z* 2087.9597 plus 54 Da of a MG-H modification). Detail glycated peptide sequence which was obtained by comparing with the theoretical data is shown in Additional file 1: Table S4. Glycated peptide has miscleavage in Arg^399^ residue. In contrast, no such peak with *m/z* value of 2141.9580 Da was found in the untreated mass spectrometry. These data suggest that MG can glycate Arg ^399^ in PKM2 of purified GAPDH complex.

**Fig. 7:**
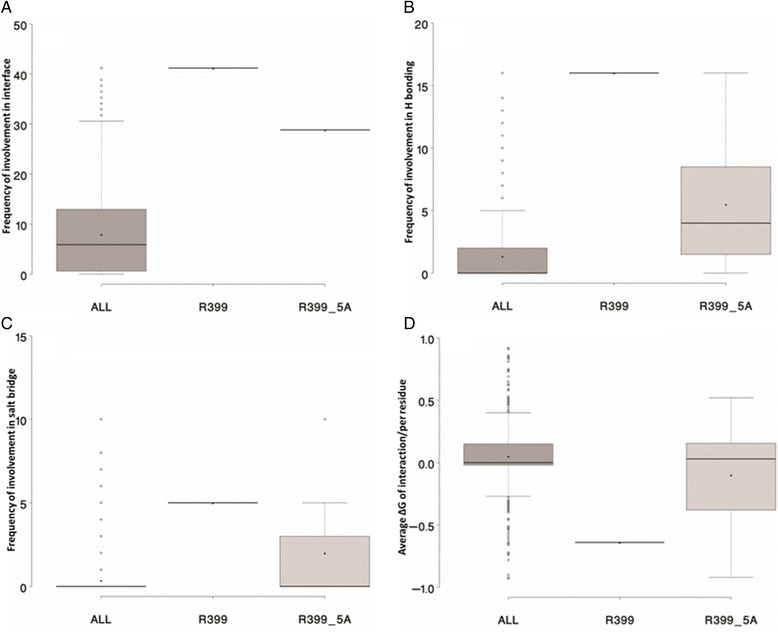
Panel **a** provides the distribution of occurrence frequencies of all residues (ALL), Arginine 399 (R399) and the residues within 5 Å distance of R399 (R399_5A) of PKM2 protein. Panel **b**, **c**, and **d** provide similar distribution of hydrogen bonding, salt bridge, and average ∆G of interaction, respectively

Previous report [34] revealed that the positively charged residue R399 of PKM2 may play a critical role in forming the PKM2 tetramer by a stable charge-charge interactions with residues E418 and E396 of PKM2 which are located on the other dimer of the tetramer PKM2. Replacing Arginine with Glutamic acid disrupts such interaction and prefers dimmer, not tetramer. When R399 was mutated with Alaline, mutant PKM2 was unable to translocate to the nucleus in EGF mediated translocation, and unable to interact with importin which facilitate nuclear translocation [35]. We assessed biochemical function of R399 residue of PKM2 using *in silico* molecular docking analysis at the interface with GAPDH. Figure 7a shows higher abundance of Arginine 399 (R399) and its neighboring residues (within 5 Å) at the interface with GAPDH. Higher frequency of hydrogen bonding (Fig. 7b) and salt bridge formation (Fig. 7c), while lower value of average ∆G of interaction (Fig. 7d) at the interface suggest the importance of R399 and its neighboring residues in the interaction with GAPDH.

## Discussion

In this paper we demonstrate that association of GAPDH with GPI or PKM2 exists in both EAC cells and 3MC induced tumor tissue. MG can glycate PKM2 at R399 residue, which may induce structural changes in GAPDH complex.

GAPDH was considered as a stable housekeeping marker for constant gene expression [36]. However, recent times have seen the ongoing discovery of new roles for GAPDH in a diverse range of cellular processes which clearly demonstrate that there are more complex roles of GAPDH in case of cellular metabolism [37, 38]. Li et al. [39] reports that GAPDH is physically associated with p300/CREB binding protein associated factor (PCAF), and histone deacetylase 5(HDAC5) for its higher enzymatic activity by acetylation at active site’s lysine 254 residue in HEK 293 T and A549 lung cancer cell line. GAPDH binds with SET protein to regulate cyclin B-cdk1 activity [40]. However we could not detect any association of these proteins with GAPDH in the cells that we have used in our study. This may be due to a different mechanism associated with post translational modification based on regulation in glycolysis pathway in different cancer cells. Interestingly, we could detect new peaks in the MG treated sample of GAPDH, GPI and PKM2 that do not have predicted m/z values. One major peak was observed in case of treated GAPDH at *m/z* value 2700 compared with untreated one (Additional file 1: Figure S6A). But, the type of modification is still unknown to us because this peak value does not match with any theoretical peptide value after addition of the mass increment due to MAGE modification. We can not rule out the possibility of such modification in GAPDH by MG, which may inhibit its enzymatic activity in cancer cells. Further studies are being carrying out in the laboratory to decipher the kind of modification occurred in the presence of MG.

Pyruvate kinase of muscle cells (PKM) exist in two isoforms: PKM1 and PKM2. They are generated due to alternative splicing [41]. PKM2 regulates many metabolic pathways for cancer cell survival and proliferation [34, 42–45]. PKM2 activity supports Warburg effect for cancer cell survival [35, 46]. We checked the expression profile of PKM isoforms in normal and 3MC induced-tumor tissues. Interestingly, PKM2 was detectable in 3MC induced tumor whereas PKM1 in normal tissue (Fig. 2a and Additional file 1: Figure S2). MG could glycate Arg^399^, which is located in alternatively spliced M2 insert of PKM2 of purified GAPDH complex. These observations led us to hypothesize that alternative splicing of PKM gene is an important factor for association of GAPDH with PKM2, which could be one of the ways for cancer cells to increase high glycolytic activity. Glycation at Arg^399^ of PKM2 followed by conformational changes of the GAPDH complexes could be one of the possible mechanisms for MG mediated inhibition of glycolysis in tumor cells.

Recently, glycolytic enzyme, GPI has been shown to regulate tumor cell growth and metastasis. Down regulation of GPI increases sensitivity towards oxidative stress and oxidative stress induced senescence [47]. Funasaka et al. [48] have shown that hypoxia increases GPI expression in human breast carcinoma BT-549 cells. Inhibition of GPI expression by siRNA reduces hypoxia induced cancer cell motility. Association of GAPDH with PKM2 and GPI may give a special advantage for cancer cell metabolism and survival. This association could be a signature for cancer cells. Further studies are needed to understand how interaction of GPI and PKM2 alters the activity of GAPDH, and helps cancer cell metabolism for developing a therapeutic approach for treatment of cancers.

## Conclusion

GAPDH interacts with an alternatively spliced isoforms of pyruvate kinase, PKM2 and GPI in cancer cells. These interactions may be one of the reasons for cancer cells to exhibit high glycolytic activity. MG can glycate at R399 residue, located in M2 insert, of PKM2 and inhibits the GAPDH activity. This could be one of the reasons for MG mediated inhibition of glycolysis in cancer cells.

## Additional file

Additional file 1: **Supplementary material.** (PDF 623 kb)

## Abbreviations

GAP: Glyceraldehyde-3-phosphate
GAPDH: Glyceraldehyde-3-phosphate dehydrogenase
GPI: Glucose-6-phosphate isomerase
IP: Immunoprecipitation
MAGE: Methylglyoxal-derived advanced glycation end-products
MALDI-TOF: Matrix-assisted laser desorption ionization-time of flight
3MC: 3-Methylcholanthrene
MG: Methylglyoxal
PKM: Pyruvate kinase muscle
PKM1: Pyruvate kinase M1
PKM2: Pyruvate kinase M2
RIPA: Radioimmunoprecipitation assay
