# Supplementary material for: Molecular association of glucose-6-phosphate isomerase and pyruvate kinase M2 with glyceraldehyde-3-phosphate dehydrogenase in cancer cells
Source: BMC Cancer. 2016 Feb 24;16:152. doi: 10.1186/s12885-016-2172-x (PMC4766697; doi:10.1186/s12885-016-2172-x)
Supplement: Supplementary file 1 — Supplementary material. (PDF 623 kb) [file 12885_2016_2172_MOESM1_ESM.pdf]

Supplementary Material

Molecular association of Glucose-6-phosphate isomerase and Pyruvate kinase M2 with Glyceraldehyde-3-phosphate dehydrogenase in cancer cells

Mahua R Das[1#], Arup K Bag[2#], Shekhar Saha[1], Alok Ghosh[3], Sumit K Dey[1], Provas Das[1], Chitra Mandal[2], Subhankar Ray[4], Saikat Chakrabarti*[5], Manju Ray*[6] and Siddhartha S Jana*[1]

Supplemental Methods:

Purification of GAPDH complex from EAC cells

GAPDH was purified from EAC cells based on its enzymatic activity which was checked after each step of purification as described previously [1]. Briefly, 30-35 grams of frozen EAC cells were homogenized in 50 mM triethanolamine-HCl buffer, pH 7.4 containing 10 mM EDTA and 10mM 2-mercaptoethanol, (referred as 'buffer A/working buffer). The supernatant was subjected to $(NH_4)_2SO_4$ fractionation steps followed by Sephacryl S-200, Sephadex G-100 (Pharmacia Fine Chemicals, Uppsala, Sweden) gel filtration columns and DEAE-Sephacell (Sigma-Aldrich, St. Louis, Missouri, USA) anion-exchange column. Enzyme fractions with high specific activity were collected and concentrated through a Amicon Ultra Centrifugal Filter Device (Ultracel-50K, Millipore Corporation, Billerica, MA, USA) and stored at -20^{0}C with preservatives for further studies.

PK assay

Pyruvate kinase (PK) catalyzes the transfer of a phosphate group from phosphoenolpyruvate (PEP) to ADP, yielding one molecule of pyruvate and one molecule of ATP. Pyruvate kinase of muscle type (PKM) has two isoforms, like PKM1 and PKM2. The enzymatic activity of both isoforms (PKM1/2) can be measured by lactate dehydrogenase (LDH) linked couple reaction where pyruvate which is produced by PKM1/2 is converted into lactate by LDH, with a concomitant oxidation of NADH to NAD+, resulting in a decrease in absorbance at 340 nm [2]. Briefly, mouse tissues (normal mice muscle and 3MC induced sarcoma) and EAC cells extracts were prepared by modified HEPES buffer (50 mM HEPES-NaOH buffer of pH 8.0, 150 mM sodium chloride, 1.0% Nonidet P-40,4 mM EDTA of pH 8.0 with freshly prepared 1 mM dithiothreitol, 0.5 mM phenylmethylsulfonyl fluoride and 1% protease inhibitor cocktails at 4^{0}C). 1 ml of assay mixture contained 10 mM HEPES-NaOH buffer of

pH 7.5, 10 mM $MgCl_{2,}$ 50mM KCl, 2 mM ADP, 1 mM PEP, 0.5 mM NADH and 0.25 unit of LDH. The reaction was started by the addition of equal amount of protein containing tissue extracts or EAC cell extract. The reaction was monitored by observing decreased in absorbance at 340 nm due to the formation of NAD^+ from NADH which was noted at 30^{-s} intervals for 2-3 min. The enzyme activity was calculated with E_{mM} = 6.22 for NADH.

Circular dichroism spectrometry

Secondary structure analysis of purified protein complex from EAC cells in the presence of MG was performed by far-UV (185-260 nm) CD in a Jasco J815 spectrophotometer at 25^0C in 1mM triethanolamine-HCl buffer, pH 7.4 containing 0.2 mM EDTA and 0.2 mM 2-mercaptoethanol using a 0.1 cm path length quartz cuvette [3]. 15 µg of purified protein complex was incubated with MG at different concentration, 0-10 mM, at 25^0C for 6h. CD spectra of the buffer was recorded and subtracted from the protein complex spectra. Each spectra line was plotted by considering the average value of three independent scans.

Table S1: List of proteins detected by mass spectroscopy analysis of band 1 of Fig. 1A.

Sr. No.	Identified Protein	Accession No.	Mascot Score
1	Pyruvate kinase, isozyme M2 (EC 2.7.1.40).- Mus musculus (Mouse)	KPYM_MOUSE	**185**
2	Pyruvate kinase muscle isozyme [Mus musculus]	CAA65761	184
3	pyruvate kinase (EC 2.7.1.40) isozyme M2 - mouse	S55921	177
4	Mus musculus 15 days embryo embryonic body below diaphragm cDNA, RIKEN full-length enriched library	Q9CRL6_MOUSE	41
5	Putative uncharacterized protein	Q9CSL2_MOUSE	38
6	Mus musculus adult female placenta cDNA, RIKEN full-length enriched library, clone:1600012K10 produ	Q9DAY3_MOUSE	34
7	Mus musculus 12 days embryo spinal cord cDNA, RIKEN full-length enriched library, clone:C530028K14	Q8BUQ3_MOUSE	34
8	Putative uncharacterized protein	Q9CTZ9_MOUSE	34
9	anti-meningococcal group C mAb C2/1076.10 immunoglobulin heavy chain [Mus musculus]	AAS47024	33
10	Immunoglobulin heavy chain VDJ region	CAC29010	30
11	Mus musculus 13 days embryo male testis cDNA, RIKEN full-length enriched library, clone:6030445D17	Q8CD43_MOUSE	29
12	Dendritic cell immunoreceptor (Mus musculus 0 day neonate cerebellum cDNA, RIKEN full-length enrich	Q9QZ15_MOUSE	29
13	Smuckler.- Mus musculus (Mouse).	Q6U7R4_MOUSE	28
14	T-cell immunoglobulin and mucin domain containing 4 Mus musculus (Mouse)	Q8CIC7_MOUSE	27
15	LOC210583 protein (Fragment).- Mus musculus (Mouse).	Q6PFY2_MOUSE	26

Table S2: List of proteins detected by mass spectroscopy analysis of band 2 of Fig. 1A

Sr. No.	Identified Protein	Accession No.	Mascot Score
1	Glucose-6-phosphate isomerase (EC 5.3.1.9) (GPI) (Phosphoglucose isomerase) (PGI)	G6PI_MOUSE	**531**
2	Glucose-6-phosphate isomerase (EC 5.3.1.9) – mouse	NUMS	528
3	Glucose phosphate isomerase 1.- Mus musculus (Mouse)	Q5RJI3_MOUSE	497
4	Phosphoglucose isomerase-2 (EC 5.3.1.9)	Q8C675_MOUSE	491
5	Glucose phosphate isomerase, partial [Mus musculus]	AAA65641	233
6	Structural glucose phosphate isomerase 1 (EC 5.3.1.9) (Fragment).- Mus musculus (Mouse)	Q9JM07_MOUSE	63
7	Jak 1 protein	Q8K0I7_MOUSE	46
8	unnamed protein product [Mus musculus]	Q8K0I7_MOUSE	42
9	JAK1 protein tyrosine kinase [Mus sp.]	AAB27517	40
10	RAC-alpha serine/threonine-protein kinase	AAA18254	39
11	protein kinase (EC 2.7.1.37) akt1 [similarity] - mouse	S33364	38
12	E230022H04Rik protein (RIKEN cDNA E230022H04 gene).- Mus musculus (Mouse)	Q8JZZ6_MOUSE	38
13	MUSHEXOKB NID: - Mus musculus	AAB57759	37
14	Mus musculus 8 days embryo whole body cDNA, RIKEN full-length enriched library, clone:5730420D13 pr	Q9CYK5_MOUSE	37
15	Dock9 protein.- Mus musculus (Mouse)	Q6PF84_MOUSE	37

Table S3: List of proteins detected by mass spectroscopy analysis of band 3 of Fig. 1A.

Sr. No.	Identified Protein	Accession No.	Mascot Score
1	Glyceraldehyde-3-phosphate dehydrogenase (phosphorylating) (EC 1.2.1.12) - mouse	DEMSG	**273**
2	Glyceraldehyde-3-phosphate dehydrogenase (EC 1.2.1.12) (GAPDH).- Mus musculus (Mouse).	G3P_MOUSE	272
3	Similar to glyceraldehyde-3-phosphate dehydrogenase.- Mus musculus (Mouse).	Q5U410_MOUSE	265
4	Glyceraldehyde-3-phosphate dehydrogenase (Phosphorylating)-like.- Mus musculus (Mouse)	Q6P287_MOUSE	175
5	Glyceraldehyde 3-phosphate dehydrogenase [Mus musculus]	AAA80276	43
6	unnamed protein product [Mus musculus]	BAB24222	35
7	Immunoglobulin heavy chain VDJ region	AAO59753	28
8	Putative uncharacterized protein [Mus musculus]	Q9D357_MOUSE	27
9	Hypothetical Protein	Q8R1N6_MOUSE	27
10	Immunoglobulin heavy chain VDJ region	AAO59739	27
11	Hypothetical protein.- Mus musculus (Mouse)	Q6P0X2_MOUSE	26
12	CQ871272 NID: - Mus musculus	CAH56817	26
13	Ig heavy chain V region (M-T408) - mouse (fragment)	S19968	26
14	ALPHA CD4 MAB IMMUNOGLOBULIN HEAVY CHAIN - VDJ REGION (FRAGMENT).- Mus musculus (Mouse)	CAA46217	26
15	MHC class Ib antigen Qa-1c.- Mus musculus (Mouse).	O98032_MOUSE	25

Table S4: Chemical identification and molecular localization of MAGE in PKM2 by MALDI-TOF analysis. Glycated residue is shown in **bold**.

Identified protein	Observed mass (Da)	Theoretical mass (Da)	Observed mass of glycated peptide (Da)	Peptide sequence	Mass Increase (Da)	Type of MAGE	Glycated residue
PKM2 Untreated	2087.9417	2088.0872	Nil	EAEAAIYHLQLFEELRR (384-400)	—	—	—
PKM2 Treated	2087.9597	2088.0872	2141.9580	EAEAAIYHLQLFEEL**R**R (384-400)	54	Hydroimidazolone	R399

Figure Legends

Fig. S1. Purification of glyceraldehydes-3-phosphate dehydrogenase (GAPDH) from EAC cells. (A) 6th step of glycolysis. (B) GAPDH activity after each step of purification. Specific activity was measured as units of activity present per mg of protein. Fold was calculated considering the specific activity of crude extract as "1". Results are expressed as means ± SD from six independent experiments. (C-E), The amino acid sequence of PKM2 (C), GPI (D) and GAPDH (E) show 21%, 45% and 24% sequence coverage respectively. Matched peptides are shown in bold red.

Fig. S2. Tissue extract from normal and 3MC induced tumor were probed with PKM1 (from Proteintech Group) and β-tubulin antibodies. Tubulin was used as loading control for samples. Note that PKM1 is absent in tumor tissue.

Fig. S3.(A) Supernatant and pellet of GAPDH -IP reaction were run along with whole tissue extract and probed with PKM2, GPI, and GAPDH antibodies. (B) Quantification of immunoblots. % of protein in the supernatant was calculated following the equation; {[S]/[S]+[P]}x100. [S] and [P] indicate the intensity of a band in supernatant and pellet, respectively. Results are expressed as means ± SD from three independent experiments.

Fig. S4: Panel A provides the average occurrence frequencies of C terminal, N terminal , and both of GAPDH and PKM2 proteins at the interface observed within the 30 top scoring docking complexes obtained from three different programs whereas panels B (GAPDH) and C (PKM2) provides the occurrence frequency of each residue within the GAPDH-PKM2 docking complexes. Panels D-F represent similar domain and residue occurrence frequencies observed within the GAPDH-GPI docking complexes. An interface is regarded as C or N terminal interface if 60% of the interface residues for each protein reside within C or N termini, respectively.

Fig. S5. (A) Enzymatic activity of PK in the extracts of normal, 3MC induced tumor tissues, and EAC cells. Note that PK activity was low in tumor cells. Results are expressed as mean ± SD from three independent experiments. **, $p<0.01$ for normal vs 3MC or EAC (B) Extract of 3MC induced tumor tissue was treated with 1mM MG or vehicle for 6 h at room temperature followed by incubation with antibody against GAPDH overnight at 4^0C. Immunoprecipitate was probed with antibodies against PKM2 (panel 1), GPI (panel 2), GAPDH (panel 3) or IgG (panel 4). Note that both GPI and PKM2 were detectable in the immunoprecipitate of GAPDH antibody in the presence of MG. (C) The circular dichroism spectroscopy of purified GAPDH protein complex from EAC cell in absence (black) and in presence of 0.5-10 mM MG at 25^0C for 6h. (D) Quantification of % of change in helicity. % was calculated following the equation: $\{[\theta]_{vehicle}-[\theta]_{sample}/[\theta]_{vehicle}\}$X100. Note that change in helicity both at 208 and 222 nm increases with increasing the concentration of MG.

Fig. S6. Glycation in GAPDH and GPI in presence of MG. Purified protein complex from EAC was treated with 1mM MG for 5 days at room temperature. Samples treated or untreated with MG were run on 7.5-15% SDS PAGE gel followed by mass spectroscopy analysis for GAPDH (A) and GPI (B). An unidentified peak is shown with arrow in (A).

References

1. Bagui S, Ray M and Ray S (1999) Glyceraldehyde-3-phosphate dehydrogenase from Ehrlich ascites carcinoma cells. Its possible role in the high glycolysis of malignant cells. *Eur J Biochem* **262**, 386-395.
2. Chern CJ, Rittenberg MB and Black JA (1972) Purification of Human Erythrocyte Pyruvate kinase. *J Biol Chem* **247**, 7173-7180.
3. Ghosh S, Ray M, Das MR, Chakrabarti A, Khan AH, Sarma DD, Acharya S (2014) Modulation of glyceraldehyde-3-phosphate dehydrogenase activity by surface functionalized quantum dots. *Phys Chem Chem Phys* **16**, 5276-83.

A

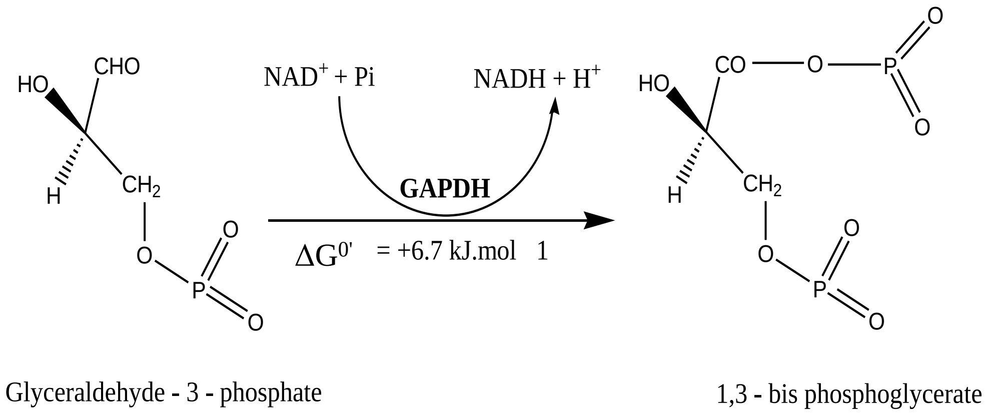
HO
CHO
H
CH2
O
P
O
O
NAD+ + Pi
NADH + H+
GAPDH
ΔG0' = +6.7 kJ.mol 1
HO
CO
O
P
O
O
H
CH2
O
P
O
O
Glyceraldehyde - 3 - phosphate
1,3 - bis phosphoglycerate

B

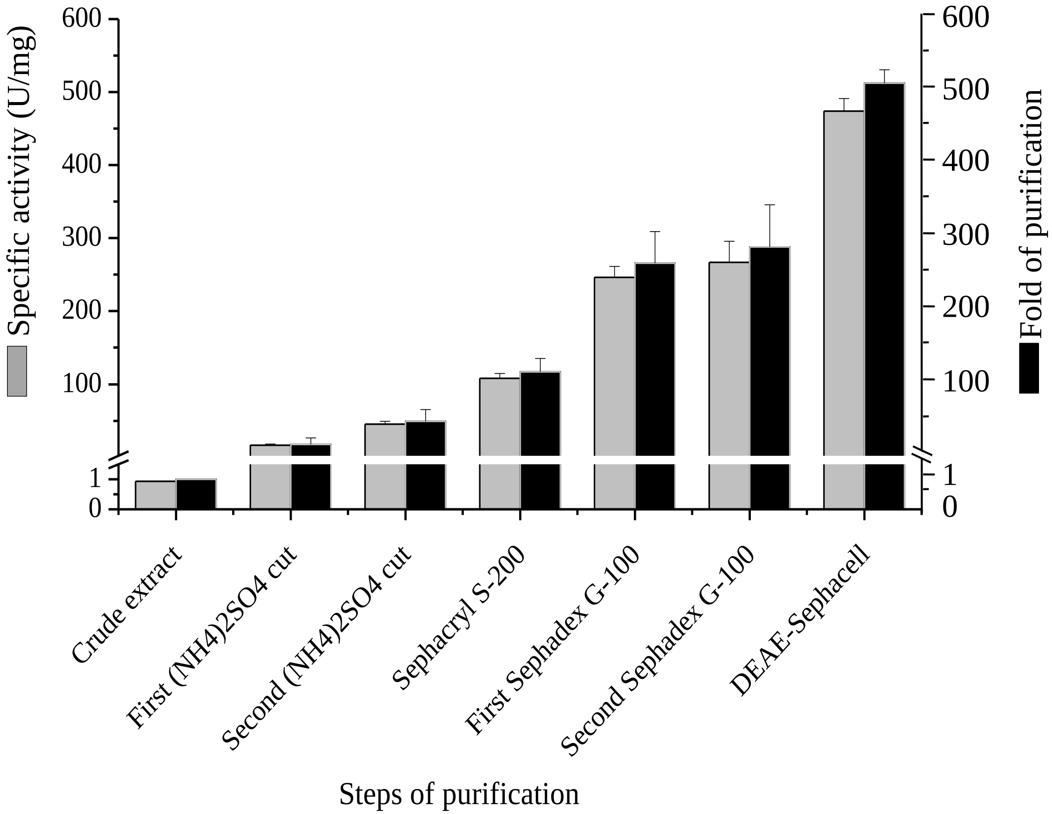
Specific activity (U/mg)
Fold of purification
600
500
400
300
200
100
1
0
Crude extract
First (NH4)2SO4 cut
Second (NH4)2SO4 cut
Sephacryl S-200
First Sephadex G-100
Second Sephadex G-100
DEAE-Sephacell
Steps of purification

Fig.S1

C

Sequence analysis of mouse PKM2 Sequence Coverage: **21%**

1	PKPHSEAGTA	FIQTQQLHAA	MADTFLEHMC	R**LDIDSAPIT**	**ARNTGIICTI**
**51**	**GPASR**SVEML	KEMIKSGMNV	ARLNFSHGTH	EYHAETIKNV	REATESFAD
**101**	PILYRPVAVA	LDTKGPEIRT	GLIKGSGTAE	VELKKGATLK	**ITLDNAYMEK**
**151**	**CDENILWLDY**	**K**NICKVVEVG	SKIYVDDGLI	SLQVKEKGAD	FLVTEVENGG
**201**	SLGSKKGVNL	PGAAVDLPAV	SEKDIQDLK**F**	**GVEQDVDMVF**	**ASFIR**K**AADV**
**251**	**HEVRK**VLGEK	GKNIKIISK**I**	**ENHEGVRRFD**	**EILEASDGIM**	**VAR**GDLGIEI
**301**	PAEKVFLAQK	MMIGRCNRAG	KPVICSTQML	EIMIKKPRPT	RAEGSDVANA
**351**	VLDGADCIML	SGETAK**GDYP**	**LEAVR**MQHLI	AREAEAAIYH	LQLFEELRRL
**401**	APITSDPTEA	AAVGAVEASF	KCCSGAIIVL	TKSGR**SAHQV**	**ARYRPR**APII
**451**	AVTRNPQTAR	QAHLYRGIFP	VLCKDAVLNA	WAEDVDLRVN	LAMDVGKARG
**501**	FFKKGDVVIV	LTGWRPGSGF	TNTMRVVPVP		

D

Sequence analysis of mouse GPI Sequence Coverage: **45%**

**1**	AALTRNPQFQ	KLLEWHRANS	ANLKLR**ELFE**	**ADPER**FNNFS	LNLNTNHGHI
**51**	LVDYSKNLVN	K**EVMQMLVEL**	**AK**SRGVEAAR	**DNMFSGSKIN**	**YTEDRAVLHV**
**101**	**ALR**NRSNTPI	K**VDGKDVMPE**	**VNR**VLDKMKS	FCQRVRSGDW	KGYTGKSITD
**151**	IINIGIGGSD	LGPLMVTEAL	KPYSKGGPR**V**	**WFVSNIDGTH**	**IAK**TLASLSP
**201**	ETSLFIIASK	TFTTQETITN	AETAK**EWFLE**	**AAK**DPSAVAK	**HFVALSTNTA**
**251**	**KVKEFGIDPQ**	**NMFEFWDWVG**	**GR**YSLWSAIG	LSIALHVGFD	HFEQLLSGAH
**301**	WMDQHFLKTP	LEK**NAPVLLA**	**LLGIWYINCY**	**GCETHALLPY**	**DQYMHRFAAY**
**351**	**FQQGDMESNG**	**K**YITKSGAR**V**	**DHQTGPIVWG**	**EPGTNGQHAF**	**YQLIHQGTKM**
**401**	**IPCDFLIPVQ**	**TQHPIRK**GLH	HK**ILLANFLA**	**QTEALMK**GKL	PEEARKELQA
**451**	AGKSPEDLEK	LLPHK**VFEGN**	**RPTNSIVFTK**	**LTPFILGALI**	**AMYEHK**IFVQ
**501**	GIMWDINSFD	QWGVELGKQL	AKKIEPELEG	SSAVTSHDSS	TNGLISFIKQ
**551**	QRDTKLE				

E

Sequence analysis of mouse GAPDH Sequence Coverage: **24%**

**1**	MVKVGVNGFG	RIGRLVTRAA	ICSGKVEIVA	INDPFIDLNY	MVYMFQYDST
**51**	HGKFNGTVKA	ENGK**LVINGK**	**PITIFQERDP**	**TNIK**WGEAGA	EYVVESTGVF
**101**	TTMEKAGAHL	KGGAK**RVIIS**	**APSADAPMFV**	**MGVNHEK**YDN	SLKIVSNASC
**151**	TTNCLAPLAK	VIHDNFGIVE	GLMTTVHAIT	ATQK**TVDGPS**	**GKLWR**DGRGA
**201**	AQNIIPASTG	AAKAVGKVIP	ELNGKLTGMA	FR**VPTPNVSV**	**VDLTCR**LEKP
**251**	AKYDDIKKVV	KQASEGPLKG	ILGYTEDQVV	SCDFNSNSHS	STFDAGAGIA
**301**	LNDNFVK**LIS**	**WYDNEYGYSN**	**R**VVDLMAYMA	SKE	

Fig.S1

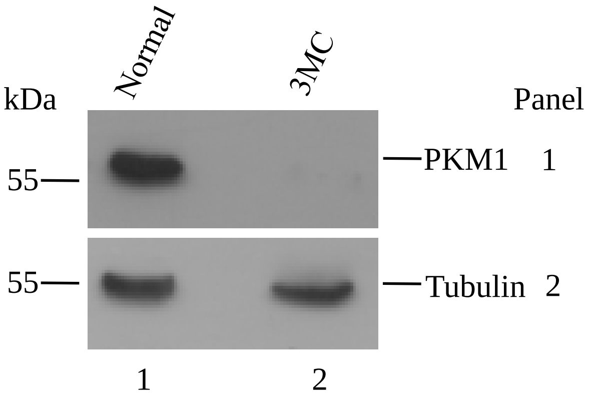

Fig.S2

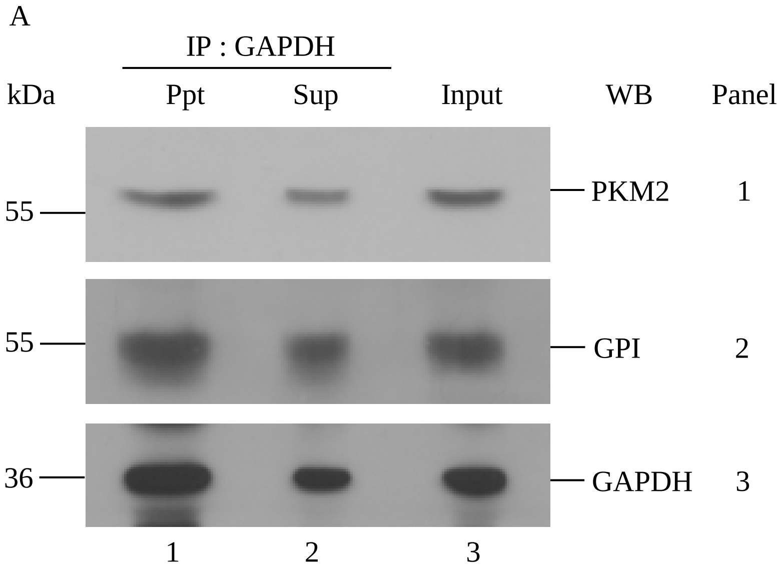
A
IP : GAPDH
kDa
Ppt
Sup
Input
WB
Panel
55
PKM2
1
55
GPI
2
36
GAPDH
3
1
2
3

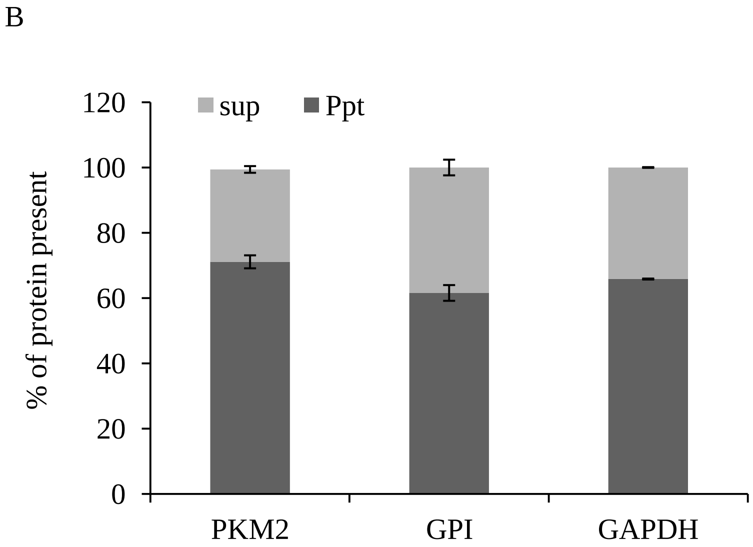
B
120
100
80
60
40
20
0
sup
Ppt
% of protein present
PKM2
GPI
GAPDH

Fig.S3

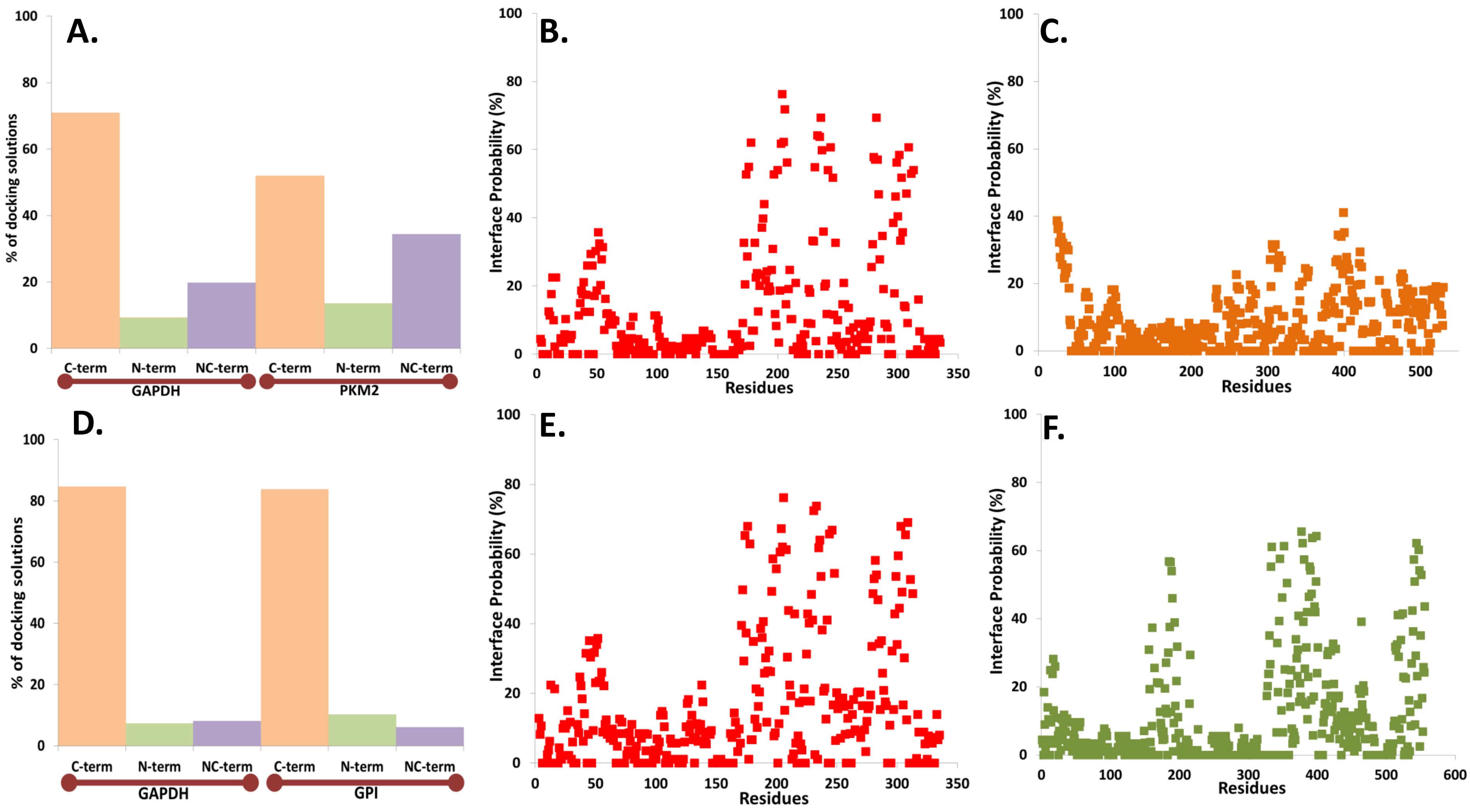
A.
B.
C.
D.
E.
F.
% of docking solutions
Interface Probability (%)
Residues
C-term
N-term
NC-term
GAPDH
PKM2
GPI

Fig.S4

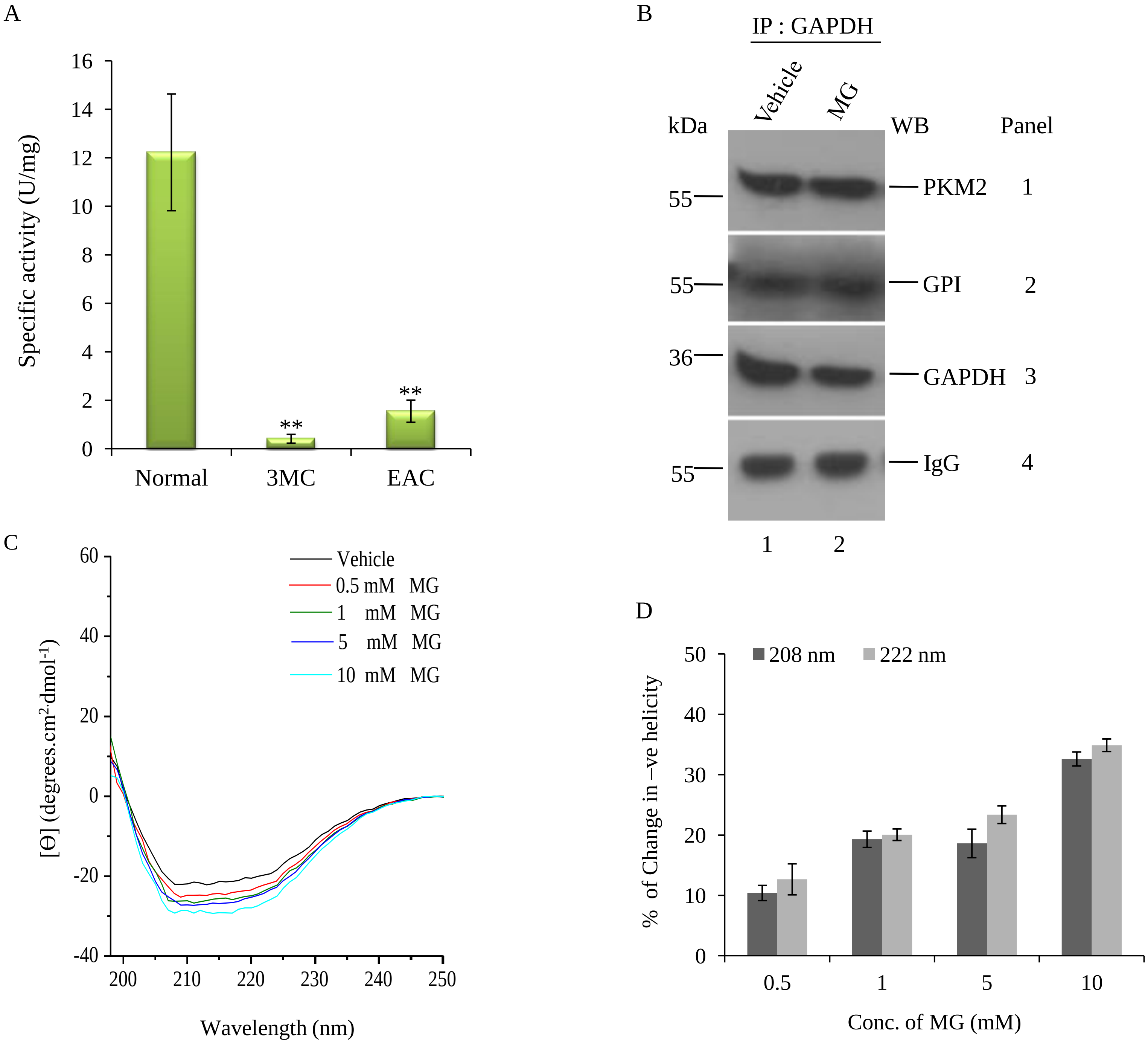
A
Specific activity (U/mg)
16
14
12
10
8
6
4
2
0
**
**
Normal
3MC
EAC
B
IP : GAPDH
Vehicle
MG
kDa
WB
Panel
55
PKM2
1
55
GPI
2
36
GAPDH
3
55
IgG
4
1
2
C
[Θ] (degrees.cm2.dmol-1)
60
40
20
0
-20
-40
Vehicle
0.5 mM MG
1 mM MG
5 mM MG
10 mM MG
200
210
220
230
240
250
Wavelength (nm)
D
% of Change in –ve helicity
50
40
30
20
10
0
208 nm
222 nm
0.5
1
5
10
Conc. of MG (mM)

Fig.S5

A

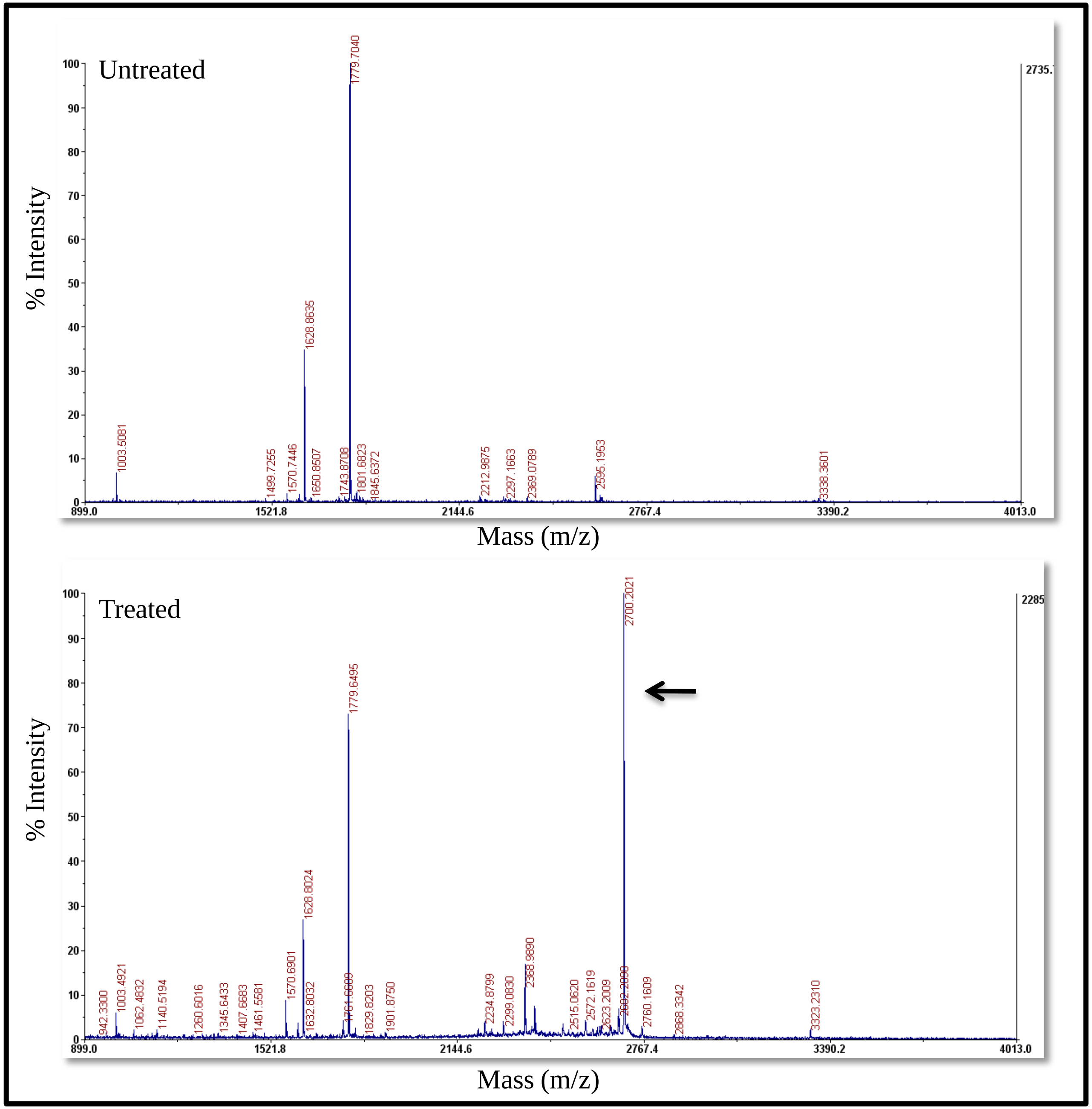

GAPDH

Fig.S6

B

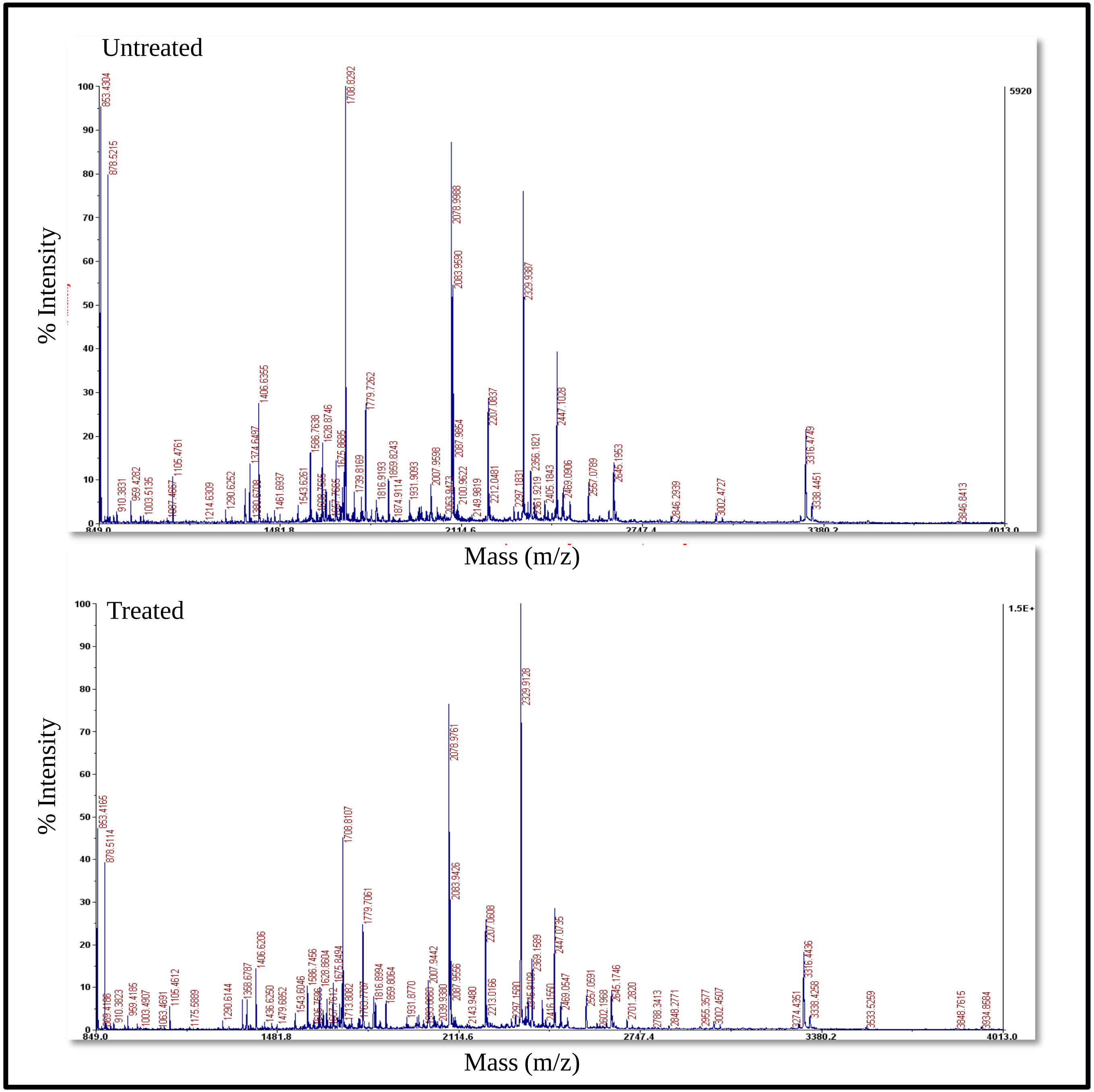
Untreated
% Intensity
Mass (m/z)
5920
853.4304
878.5215
1708.8292
2078.9968
2083.9590
2329.9387
2447.1028
1406.6355
1779.7262
3316.4749
Treated
% Intensity
Mass (m/z)
1.5E+
2329.9128
2078.9761
2083.9426
1708.8107
1779.7061
853.4165
878.5114
849.0
1481.8
2114.6
2747.4
3380.2
4013.0

GPI

Fig.S6
